# I'll Show You Mine, If You Show Me Yours

**DOI:** 10.1100/tsw.2001.7

**Published:** 2001-02-16

## Abstract

The human genome dominated science news last week. Both Science and Nature lead this week with articles about the simultaneous publication of the human genome sequence by the private company Celera Genomics and the publicly funded Human Genome Project (HGP).

The often bitter and contentious race between Celera and HGP to complete sequencing the human genome ended in a tie last week, report both *Science* and *Nature*. Celera published its sequence in *Science* and HGP published their results in *Nature*. But the term "publish" must be taken with a grain of salt. Only HGP has actually made its genome sequence publicly available. The HGP version will be deposited in the public database GenBank. Interested scientists will have to pay for access to the privately held Celera version. And whether or not geneticists have anything to gain by paying Celera was hotly debated at news conferences around the globe, reports *Nature*. Although Celera scientists had often criticized the HGP method as being inefficient, HGP now argues that without the extra information they generated, Celera would not have been able to complete its sequence. In *Nature*, HGP team member John Sulston, former director of Britain's Sanger Centre, said, "We have saved the day. They [Celera] failed by their own standards."

Celera acknowledges using the free HGP data in *Nature*, but Gene Myers, now vice-president of informatics research with Celera, dismissed the criticisms from the HGP members as "pure speculation." Celera President Craig Venter did, however, acknowledge that the free HGP data was useful. In *Nature*, Venter says that his company decided to make use of the public data "instead of spending six months and [another] $60 million" to assemble their sequence. *Science* largely ignored the dispute in its lead story, despite the fact that the journal played a very visible role in the argument. Their decision to publish the Celera paper without requiring the company to make their data available has drawn heavy criticism from the scientific community in the past weeks.

Instead, *Science*'s lead story on the genome chose to concentrate on several new discoveries. Chief among these was the realization that the human genome has a mere 32,000 genes, or about twice as many as a nematode, reports *Science*. In *Science*, Frances Collins, director of the National Human Genome Research Institute, says the surprisingly low number "is a bit of an assault on our sensibility." The primary difference between a nematode and a human, according to scientists quoted in *Science*, is not the number of genes, but how hard they work. Humans squeeze more information out of each gene.

But as exciting as the first discoveries are, everyone agrees that the real work has only just begun. In *Science*, Fred Sanger, a retired pioneer in gene sequencing, says, "It's like a book in a foreign language that you don't understand. That's the first job, working the language out."
